# A perspective of lipid nanoparticles for RNA delivery

**DOI:** 10.1002/EXP.20230147

**Published:** 2024-04-15

**Authors:** Yutian Ma, Shiyao Li, Xin Lin, Yupeng Chen

**Affiliations:** ^1^ Division of Pharmacoengineering and Molecular Pharmaceutics, Eshelman School of Pharmacy University of North Carolina at Chapel Hill Chapel Hill North Carolina USA; ^2^ School of Science RMIT University Bundoora Victoria Australia; ^3^ ARC Centre of Excellence in Convergent Bio‐Nano Science and Technology, and the Department of Chemical Engineering The University of Melbourne Parkville Victoria Australia; ^4^ Department of Cell Biology Duke University Medical Center Durham North Carolina USA; ^5^ CAS Key Laboratory for Biomedical Effects of Nanomaterials and Nanosafety, CAS Center for Excellence in Nanoscience National Center for Nanoscience and Technology Beijing China; ^6^ University of Chinese Academy of Sciences Beijing China

**Keywords:** biomolecules, bionanomaterials, COVID‐19, lipid nanoparticles, RNA delivery platform

## Abstract

Over the last two decades, lipid nanoparticles (LNPs) have evolved as an effective biocompatible and biodegradable RNA delivery platform in the fields of nanomedicine, biotechnology, and drug delivery. They are novel bionanomaterials that can be used to encapsulate a wide range of biomolecules, such as mRNA, as demonstrated by the current successes of COVID‐19 mRNA vaccines. Therefore, it is important to provide a perspective on LNPs for RNA delivery, which further offers useful guidance for researchers who want to work in the RNA‐based LNP field. This perspective first summarizes the approaches for the preparation of LNPs, followed by the introduction of the key characterization parameters. Then, the in vitro cell experiments to study LNP performance, including cell selection, cell viability, cellular association/uptake, endosomal escape, and their efficacy, were summarized. Finally, the in vivo animal experiments in the aspects of animal selection, administration, dosing and safety, and their therapeutic efficacy were discussed. The authors hope this perspective can offer valuable guidance to researchers who enter the field of RNA‐based LNPs and help them understand the crucial parameters that RNA‐based LNPs demand.

## Graphical abstract

This perspective summarizes the approaches for the preparation of lipid nanoparticles (LNPs), the key characterization parameters, in vitro cell experiments, and in vivo animal experiments. The authors hope this perspective can offer valuable guidance to researchers who enter the field of RNA‐based LNPs and help them understand the crucial parameters that RNA‐based LNPs demand.

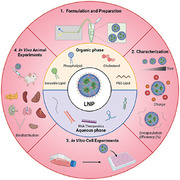

## 1 INTRODUCTION

Over the past two decades, the development of gene therapies, especially RNA‐based therapies, has emerged as a groundbreaking frontier, offering promising solutions to treat various diseases, including genetic disorders, cancers, neurodegenerative disorders like sensorineural hearing loss, and infection diseases.^[^ ^1^, ^2^, ^3^, ^4^, ^5^, ^6^, ^7^, ^8^, ^9^, ^10^ ^]^ Recent examples of RNA‐based therapies approved by the U.S. Food and Drug Administration (FDA) include ONPATTRO (patisiran) for delivering the double‐stranded small interfering RNA (siRNA) to treat polyneuropathy and cardiomyopathy and messenger RNA (mRNA) COVID‐19 vaccines made by Moderna or Pfizer/BioNTech.^[^ ^11^, ^12^, ^13^, ^14^, ^15^, ^16^, ^17^ ^]^ The central success of these RNA‐based therapies is to deliver RNA therapeutics to target cells using lipid nanoparticles (LNPs). LNPs have risen as a powerful and versatile tool for drug delivery due to their high biocompatibility and biodegradability. They can not only protect RNA from degradation but also facilitate their uptake and further delivery of RNA therapeutics into the cytosol of the target cells. They typically consist of four different components: DLin‐MC3‐DMA (ONPATTRO)/SM‐102 (Moderna)/ALC‐0315 (BioNTech/Pfizer) as the ionizable lipid, DSPC as the phospholipid and PEG‐2000‐C‐DMG (ONPATTRO)/DMG‐PEG‐2000 (Moderna)/ALC‐0159 (BioNTech/Pfizer) as the PEG‐lipid and cholesterol.^[^ ^18^, ^19^, ^20^, ^21^, ^22^ ^]^

Ionizable lipids typically contain tertiary amine groups that can be deprotonated at neural pH but protonated when the pH is below their pKa.^[^ ^23^ ^]^ The main functions of ionizable lipids are to encapsulate RNA therapeutic drugs in LNPs and potentially facilitate their endosomal escape. Phospholipids, as a type of helper lipid, are to support the stability of LNP during storage and circulation. PEG‐lipids are mainly related to preventing LNP aggregation and enhancing stability during their formulation and storage. They might also affect other properties such as RNA encapsulation efficiency (EE), circulation time, transfection efficiency, and immune response.^[^ ^24^, ^25^, ^26^ ^]^ The main function of cholesterol is to improve the circulation time and maintain the integrity of LNPs. A detailed explanation of the functions of each LNP component has been summarized elsewhere.^[^ ^27^ ^]^

**FIGURE 1:**
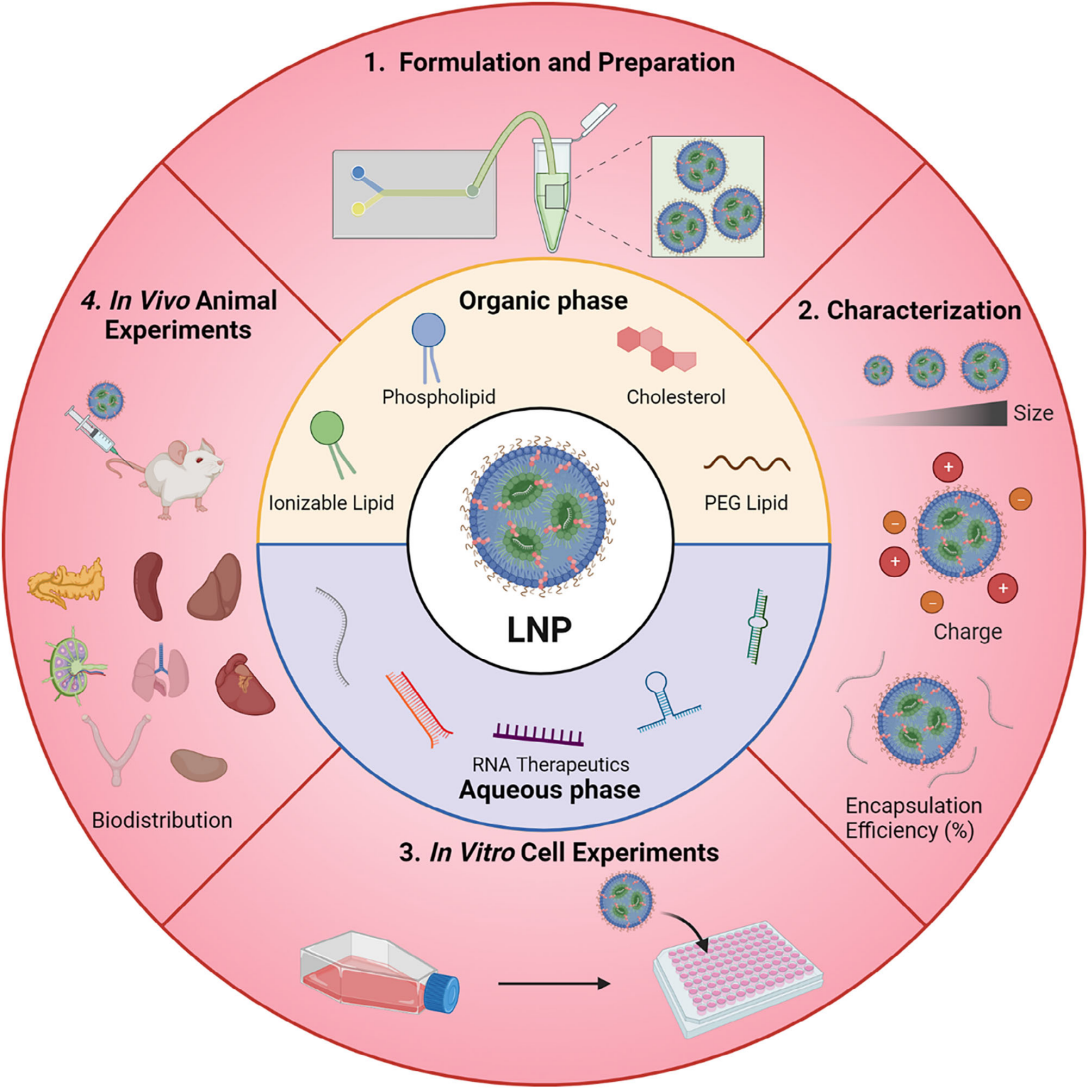
Evaluation of lipid nanoparticles (LNPs) includes LNP formulation and preparation, characterization, in vitro cell experiments and in vivo animal experiments.

In recent years, many reviews or perspectives about RNA‐based LNPs have largely focused on how RNA elicits biological changes and their potential therapeutic applications. By contrast, we provide a perspective on studying RNA‐based LNPs (Figure 1). We first start with the methods of LNP formulation and preparation. Then we discuss the proper characterization of LNPs, including their size, polydispersity index (PDI), charge, surface morphology, RNA therapeutic encapsulation efficiency (EE), and stability. Subsequently, we focus on the in vitro cell experiments, including cell selection, cell viability, cellular association/uptake, endosomal escape, and their efficacy. Finally, we investigate in vivo animal experiments to study the LNP performance in the aspects of animal selection, administration, dosing and safety, and their therapeutic efficacy. We aim to provide a comprehensive overview for investigating RNA‐based LNPs, which can provide useful guidance for researchers who want to work in the RNA‐based LNP field.

## 2 EVALUAITON OF LNP

### 2.1 LNP formulation and preparation

LNP, as a versatile drug delivery platform, can encapsulate various therapeutic drugs, including small‐molecule drugs, proteins, and RNA drugs.^[^ ^28^ ^]^ The structures of each LNP component can be easily modified to change their physico‐chemical properties, such as solubility, stability, and bioactivity, and the release profiles of therapeutic drugs into the target organs. Several formulation techniques have been employed to formulate LNPs. One method of engineering LNPs is the solvent‐based emulsification method, including emulsion‐solvent evaporation, solvent injection, and solvent displacement.^[^ ^29^, ^30^ ^]^ Specifically, this method involves the dissolution of lipids and therapeutic drugs in a water‐immiscible organic solvent such as toluene and chloroform, followed by dispersing this solution into an aqueous phase such as water to form the oil‐in‐water emulsions.^[^ ^31^ ^]^ The LNPs are formulated after the evaporation of the organic solvents, which can be used for encapsulating temperature‐sensitive therapeutic drugs. Therefore, it is a versatile and straightforward method that is highly reproducible and can potentially scale up for industry production. However, the complete removal of organic solvent might be difficult, possibly causing some toxicity issues, and the need for solvent removal might require further complicated steps.^[^ ^29^, ^30^, ^32^ ^]^ Moreover, it is difficult to formulate LNP with a high loading capacity using this method, especially for hydrophilic drugs such as RNA therapeutics.

The nonsolvent‐based emulsification method involves melted lipids for the formulation of oil‐in‐water emulsions.^[^ ^33^ ^]^ Specifically, solid lipids are required to melt into liquid, followed by mixing the lipids with a surfactant solution in the aqueous phase using several approaches, like high‐pressure homogenization, high‐speed stirring or ultrasonication, microemulsions, and membrane emulsification.^[^ ^34^, ^35^, ^36^, ^37^, ^38^ ^]^ Then the LNPs are obtained by cooling them in an ice bath.^[^ ^39^ ^]^ The quality of formulated LNPs relies on several parameters, such as lipid or surfactant type, lipid or surfactant concentration, and homogenization or sonication time.^[^ ^40^ ^]^ The advantage of using this technique is to avoid using toxic organic solvents, facilitating their biological applications. However, this technique requires specialized equipment and is not suitable for temperature‐sensitive biomolecules, as the high melting temperature of lipids might affect their stability.^[^ ^41^ ^]^

Microfluidic mixing is an advanced technique that employs precisely controlled flow conditions in microfluidic devices to formulate LNPs. Microfluidic devices can typically be classified into chip‐based microfluidic devices such as hydrodynamic flow focusing (HFF) design, a tesla‐structure design, or staggered herringbone micromixer (SHM) structure design and capillary‐based microfluidic devices.^[^ ^42^, ^43^, ^44^, ^45^, ^46^ ^]^ These devices enable exceptional reproducibility and control over particle size with a narrow size distribution and drug EE.^[^ ^47^ ^]^ For instance, researchers have compared the quality of siRNA‐LNPs using a microfluidic device and vortex mixing.^[^ ^48^ ^]^ They found that siRNA‐LNPs made using microfluidic devices had an average size of 38 nm with a narrower size distribution and a 20% EE promotion compared to the LNPs prepared via vortex mixing. Building on those advantages, microfluidic mixing devices are the most commonly used technique to prepare RNA‐loaded LNPs with ionizable lipids, helper lipids, cholesterol, and PEG lipid. Other LNP formulation approaches, such as bulk nanoprecipitation, coacervation method, supercritical fluid technology and large‐scale production, can also be utilized for making LNPs, which has been summarized elsewhere.^[^ ^49^ ^]^

In summary, the formulation of LNPs is a dynamic field with a range of techniques available to create tailored drug delivery systems. The choice of formulation method depends on the specific drug and application requirements, with an ongoing emphasis on enhancing drug encapsulation, stability, and controlled therapeutic release to optimize therapeutic outcomes.

### 2.2 Characterization

**FIGURE 2:**
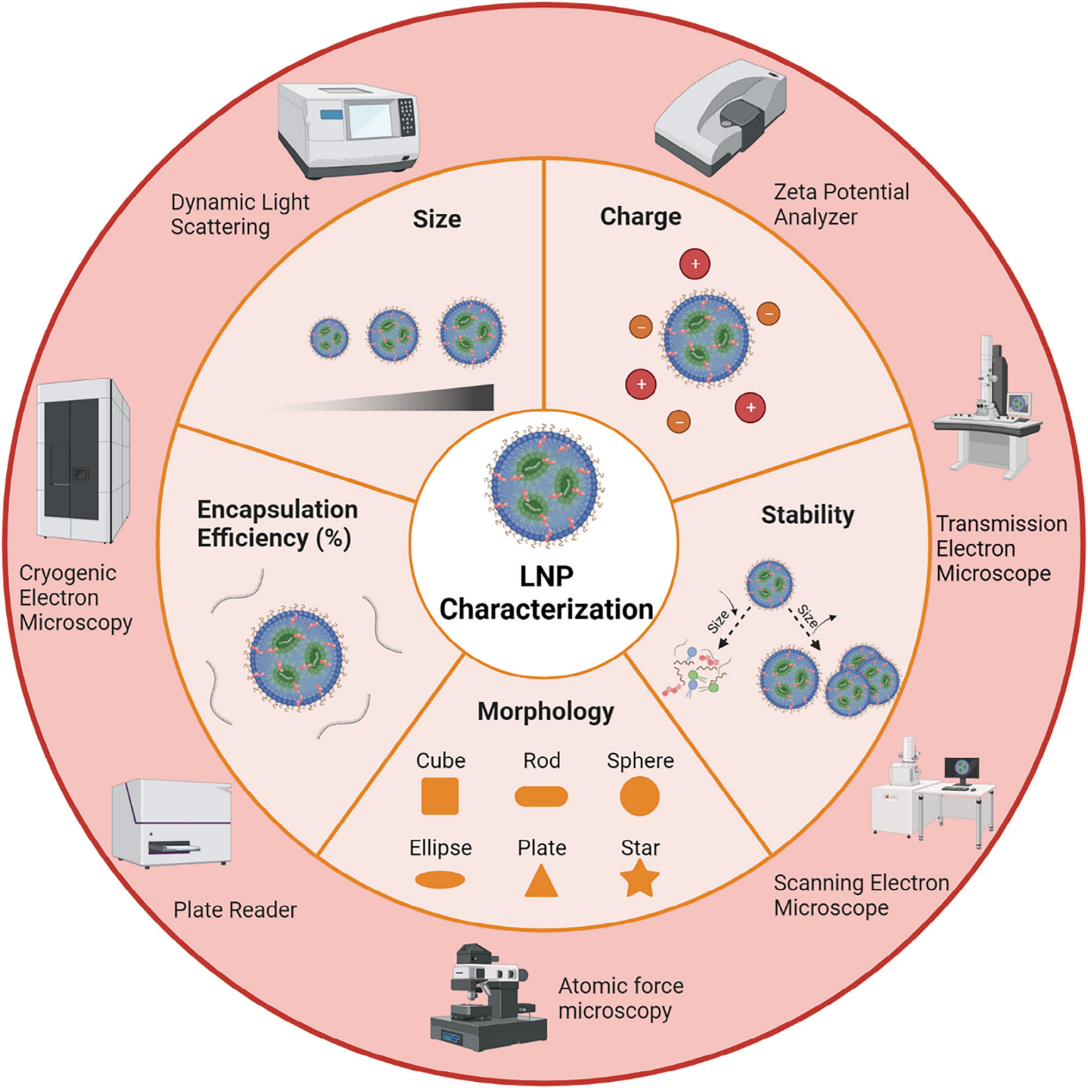
Key characterization parameters of LNPs with some representative characterization instruments.

To ensure the quality and effectiveness of LNPs, several commonly used parameters are employed, such as size, PDI, charge, surface morphology, RNA therapeutics EE, and stability (Figure 2). Typically, the average size of LNPs ranges from 100 to 400 nm, and LNPs with a PDI value less than 0.3 are considered as good LNPs for most of the studies.^[^ ^49^, ^50^ ^]^ A smaller PDI indicates a narrow size distribution, which may lead to less variability for their in vitro and in vivo performance. The size and PDI can be determined by dynamic light scatting (DLS), which can detect the different fluctuating light intensities related to the Brownian motion of LNPs.^[^ ^51^, ^52^ ^]^ Charges of LNPs may potentially influence their stability and are typically measured by a zeta potential analyzer. For RNA‐based LNPs, typically a near‐neutral charge is preferable.^[^ ^53^ ^]^ The neutral charge of LNPs not only ensures their stability in the biological environment, which is crucial for maintaining the integrity of LNPs until they reach their target site, but it also leads to lower immunogenicity and reduced clearance by the reticuloendothelial systems such as splenic macrophages and Kupfer cells.^[^ ^53^ ^]^ Additionally, positively charged nanoparticles typically result in high cytotoxicity due to the high percentage of internalization.^[^ ^53^, ^54^, ^55^ ^]^ Electron microscopy, including transmission electron microscopy (TEM), cryogenic electron microscopy (Cyro‐EM), and scanning electron microscopy (SEM), can also be used to characterize the size and the morphology/shape of LNPs.^[^ ^56^ ^]^ These are also important parameters since some studies have suggested that the shape of LNPs could affect their transfection efficiency.^[^ ^57^ ^]^ Moreover, atomic force microscopy (AFM) can be employed to characterize their thickness and generate a 3D structure of LNPs. Further, stability is important for the long‐term storage and viability of LNPs. Storage stability, including temperature (such as 4°C, room temperature, −20°C or −80°C), time (from days to weeks), and resistance ability to environmental changes such as pH (e.g. pH 4, 6, and 7.4), cell culture media (e.g. DMEM, RPMI), human plasma or serum, and freeze‐thaw cycles, can also be tested to ensure the effectiveness of LNPs during storage and administration.

RNA EE is another important parameter to evaluate the performance of LNPs. Generally, the RNA encapsulation can be measured by the Ribogreen assay.^[^ ^58^ ^]^ Specifically, the RNA concentration outside LNPs is first measured. Then the overall RNA concentration of both inside and outside of LNPs is measured by dissolving the LNPs using some surfactants, such as Triton. Then the EE and the overall mRNA concentration can be calculated using the following formula:

$$\%\mathrm{EE}=\left(1-\frac{\text{RNA concentration outside LNPs}}{\text{Overall RNA concentration}}\right)\times 100\%$$

Normally, higher EE might be desirable, as it provides the possibility of delivering more RNA therapeutics to target cells.^[^ ^59^ ^]^

### 2.3 In vitro cell experiments

#### 2.3.1 Cell culture and viability

**FIGURE 3:**
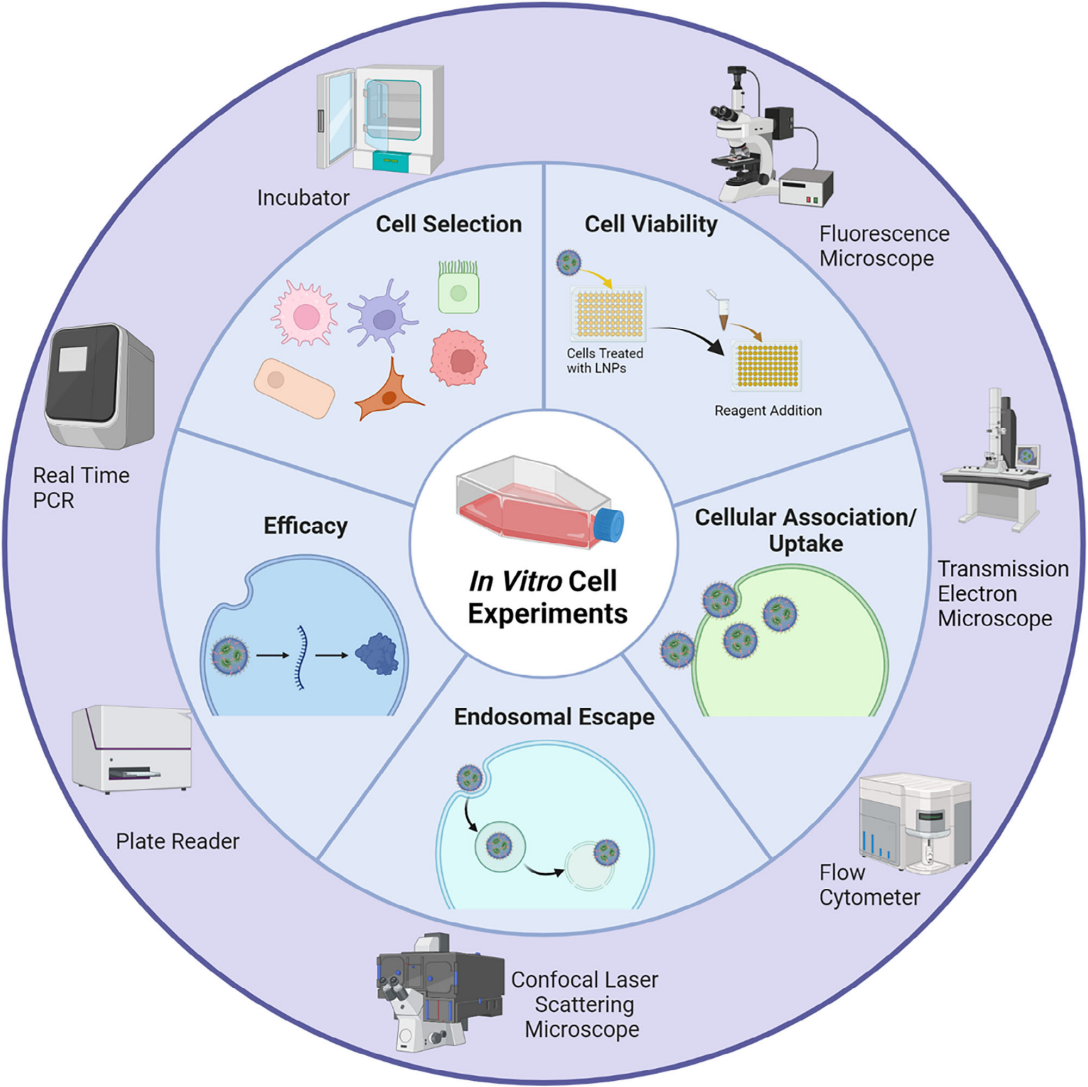
Key in vitro assessment parameters of RNA based LNPs and with their representative characterization instruments.

RNA‐based LNPs have evolved as a versatile platform for a wide range of biomedical applications, such as vaccines, cancer therapies, and regenerative medicines. The in vitro evaluation of RNA‐based LNPs generally involves several aspects such as cell selection, cell viability and in vitro efficacy, and mechanism assessments including their cellular association/uptake and endosomal escape (Figure 3). To evaluate their activity or efficacy, the first thing is to select appropriate cells for RNA‐based LNPs, as the choice of cells should align with the specific objectives and the intended application of LNP systems. There are several considerations for selecting appropriate cells for in vitro RNA‐based LNP studies. First, selected cells should mimic the target tissues or organs. For example, if researchers want to develop LNPs mainly for brain glioblastoma cancer therapies, U87 and U251 cells may be suitable selections.^[^ ^60^ ^]^ If developed LNPs are mainly for vaccine application, dendritic cells such as DC 2.4 may be a good choice. Second, selected cells should be aligned with the target organs of developed LNPs. For instance, if developed LNPs mainly express proteins in the liver, HepG2 and Huh‐7 cells may be suitable selections.^[^ ^61^ ^]^ Third, it is also suggested to consider cell growth characterizations such as cell division speed, and culture requirements like media and pH.

Upon selecting the cells, it is important to assess the in vitro cell viability of RNA‐based LNPs, which is crucial for understanding the safety and efficacy of the delivery system when exposed to biological environments. Researchers can determine the impact of LNPs on cell viability, enabling the evaluation of biocompatibility and the customization of designs for diverse biomedical applications. Moreover, the study of cell viability aids in assessing the efficacy of LNPs, since high toxicity in the developed LNP systems could be a potential cause for the very low transfection efficiency. Hence, it is necessary to simultaneously perform cell viability assessments during transfection efficacy experiments. Additionally, understanding cell viability can play a vital role in minimizing potential health risks associated with developed RNA‐based LNP systems.

Cell viability studies can be conducted through various approaches. Among these, several cell viability assays are employed to assess the tolerability of RNA‐based LNPs. These include the MTT/XTT assay, which measures mitochondrial activity; the Alamar Blue assay which utilizes resazurin‐based reagents to gauge cell metabolic activity, the lactate dehydrogenase (LDH) assay for assessing cell membrane integrity; and the CellTiter‐Fluor assay, which measures conserved and constitutive protease activity. These assays collectively allow the evaluation of the potential cytotoxic effects of LNPs on the selected cells. Second, dose‐responsive studies can also be performed to evaluate the cytotoxicity of the RNA‐based LNPs to determine their minimal cytotoxicity, or IC50, ensuring the effective delivery of RNA drugs. Third, time‐dependent studies are required to evaluate how cell viability is affected by the LNP treatment, which is essential to evaluate the long‐term impact or stability of the RNA‐based LNP platforms. Fourth, it is also suggested to include the positive control, such as lipofectamine or FDA approved DLin‐MC3‐DMA, SM‐102, and ALC‐0315 LNPs as a comparison with the developed LNP system, and the kill control (e.g. membrane surfactants like triton or sodium dodecyl sulfate (SDS)), to validate the cytotoxicity assay working properly. Fifth, it is also vital to use live/dead cell stain assays to visualize the cytotoxicity of the LNP system as a further confirmation of cell viability, which can be achieved by fluorescent microscopy. Taken together, selecting the correct cells and conducting thorough viability studies will provide valuable insights into the safety and effectiveness of RNA‐based LNP systems, helping to advance the development of innovative RNA therapeutics.

#### 2.3.2 Cellular association/uptake

LNPs can interact with cells at both cellular and molecular levels. At the cellular level, LNPs interact with cell membranes, whose internalization is influenced by the surface charge, size, and lipid composition of the LNPs. Once inside the cells, they were internalized into the endosomes, thereby requiring strategies for efficient endosomal escape to ensure the release of RNA drugs into the cytoplasm. At the molecular level, LNPs can modulate gene expression by delivering nucleic acids, such as mRNA or siRNA, to the cellular machinery, further achieving their therapeutic applications. Understanding these cellular and molecular interactions provides the fundamental criteria for optimizing LNP designs, enhancing drug delivery efficiency, and minimizing off‐target cytotoxic effects, thereby advancing the development of effective and targeted therapeutic interventions.

Cellular association and cellular uptake refer to the LNP interacting with the outer surface of a cell or being internalized by a cell, respectively, which are essential for drug delivery and gene therapies. Cell association is the first step in the process of cellular uptake. It relies on the physico‐chemical properties of LNPs, like lipid composition or excipients, and the specific characteristics of the cell type. Once LNPs attach to the surface of the cells, they will then enter the cells via several pathways, such as endocytosis (including phagocytosis and pinocytosis) and direct fusion with the cell membranes.^[^ ^62^ ^]^ Therefore, understanding the predominant mechanism can guide the design of RNA‐based LNPs tailored for specific cell types. Moreover, studying the cellular association and uptake of RNA‐based LNP systems is particularly important to optimize their delivery to particular tissues or organs. It can also provide important information to optimize/design LNP systems for enhancing specific cell targeting and reducing non‐specific cell binding.

To evaluate the cellular association/uptake, fluorescently labeled LNPs are typically employed, allowing researchers to visualize their association with cells under confocal or super‐resolution microscopy (e.g. stochastic optical reconstruction microscopy (STORM) and super‐resolution structured illuminated microscopy (SR‐SIM)) or quantify it through flow cytometry.^[^ ^63^, ^64^ ^]^ The percentage of the cellular association or uptake depends on the composition, size, and surface charge of LNPs, cell type, and the presence of serum proteins. Taken in tandem, understanding how LNPs interact with cells at the cellular and molecular level is critical for the design of more effective delivery vehicles for RNA drugs.

#### 2.3.3 Endosomal escape

After internalization by cells, LNPs often enter endosomal compartments. Endosomes can entrap them inside without releasing their RNA cargo into the cell's cytoplasm, affecting their therapeutic efficacy. Some estimates show that <2% of RNA LNP dose can escape endosomal compartments.^[^ ^65^, ^66^ ^]^ Therefore, it is crucial to evaluate the endosomal escape ability of LNPs and develop diverse strategies, such as optimizing LNP formulation, designing LNPs to respond to the endosomal environment, or adding excipients such as tannic acid, facilitating efficient endosomal escape.^[^ ^67^, ^68^, ^69^ ^]^ Moreover, the improvement of the endosomal escape capability can also minimize the required RNA therapeutic doses, thus reducing their off‐target side effects.

There have been several mechanisms proposed to facilitate endosomal escape. Proton sponge effect is one of the possible mechanisms. This mechanism is based on the influx of protons into endosomes after the internalization of LNPs, which increases the osmotic pressure, and subsequently, release the LNPs into cytosol.^[^ ^70^ ^]^ Moreover, It is believed that optimization of the ion transporter across plasma membranes might also potentially do favors for altering ion homeostasis to mediate the release of LNPs.^[^ ^71^, ^72^ ^]^ Endosomal membrane fusion is another potential mechanism in which lipid‐based systems will fuse directly with the endosomal membrane, allowing them to bypass endosomal compartments and enter the cytoplasm. In addition, LNPs with modification of pH‐sensitive lipids might also disrupt the endosomal membrane at lower pH levels (environment of the endosomes). On the other hand, some studies also suggest that the mechanism of endosome escape is due to the formulation of multiple transient pores over time, as lysosomal pH remains unchanged.^[^ ^73^, ^74^ ^]^ Therefore, understanding the exact mechanism and enhancing endosomal escape is a critical aspect of developing effective RNA‐based LNPs. The detailed endosomal escape mechanism of nanoparticles has been summarized elsewhere.^[^ ^75^ ^]^

Several techniques and methods are commonly used to assess their endosomal escape capabilities. First, endosomes can be fluorescently labeled with fluorescent dyes (e.g. Lysotracker) or tagged proteins, and LNPs or RNA are fluorescently labeled with another fluorescent dyes, then the co‐localization can be visualized using fluorescent microscopy, confocal microscopy or super‐resolution microscopy.^[^ ^76^ ^]^ The endosomal capabilities can be quantified using Pearson's correlation coefficient (PCC), where a coefficient with a value between 0 and 1 (where 0 suggests complete endosomal escape and 1 suggests no endosomal escape).^[^ ^67^, ^77^, ^78^, ^79^ ^]^ Second, a membrane impermeable dye, calcein leakage assay is another strategy to evaluate the endosomal escape capability of LNPs, in which punctuated fluorescent dots are observed when entrapped inside the endosomes but become distributed throughout cells if endosomal membranes ruptured.^[^ ^67^, ^80^ ^]^ Third, endosomal escape capabilities can be quantified using quantitative PCR techniques for the measurement of RNA drugs in the cytoplasm. Fourth, electron microscopy such as transmission electron microscopy (TEM) is another useful strategy to visualize the ultrastructure of cells and display the presence of LNPs within endosomes or in the cytoplasm.^[^ ^81^ ^]^ Fifth, gold nanoparticles can also potentially be used as markers for tracking the intracellular localization of LNPs and whether they have escaped from endosomes.^[^ ^82^, ^83^ ^]^ Researchers often use a combination of those methods to get a comprehensive understanding of endosomal escape by RNA‐based LNPs. The choice of technique depends on the specific experimental goals, the availability of equipment and expertise, and the nature of the LNP system being studied.

#### 2.3.4 In vitro efficacy

Upon escape from endosomes, RNA therapeutic drugs should be released into the cytoplasm and achieve their therapeutic functions. An easy and pilot study is to encapsulate firefly luciferase (FLuc) or enhanced green fluorescent protein (EGFP)‐coded mRNA and evaluate their transfection efficiency on cultured cells, because quantification of these two upregulated proteins is easy and reliable. There are quite a few assays that can quantify the FLuc protein expression level, such as the Bright‐Glo luciferase assay, the Dual‐Luciferase reporter assay, and the Steady‐Glo luciferase assay.^[^ ^84^, ^85^, ^86^ ^]^ The EGFP protein signals can be easily quantified using a plate reader. They can also easily visualize under a fluorescent microscope. Other mRNA, such as human erythropoietin (EPO) mRNA, can also be used as a pilot study to evaluate the secreted protein level of LNP systems. Similar to cell viability assessment, dose‐responsive and time‐dependent studies can also be performed to evaluate the in vitro transfection efficacy of mRNA‐based LNPs. Those experiments can be used to screen a library of LNP systems, facilitating researchers to select the best performance LNP platforms with further encapsulation of their target genes. In summary, assessing the in vitro performance and efficacy of LNPs can provide insights and predict their ability in vivo.

### 2.4 In vivo animal experiments

#### 2.4.1 Animal selection

**FIGURE 4:**
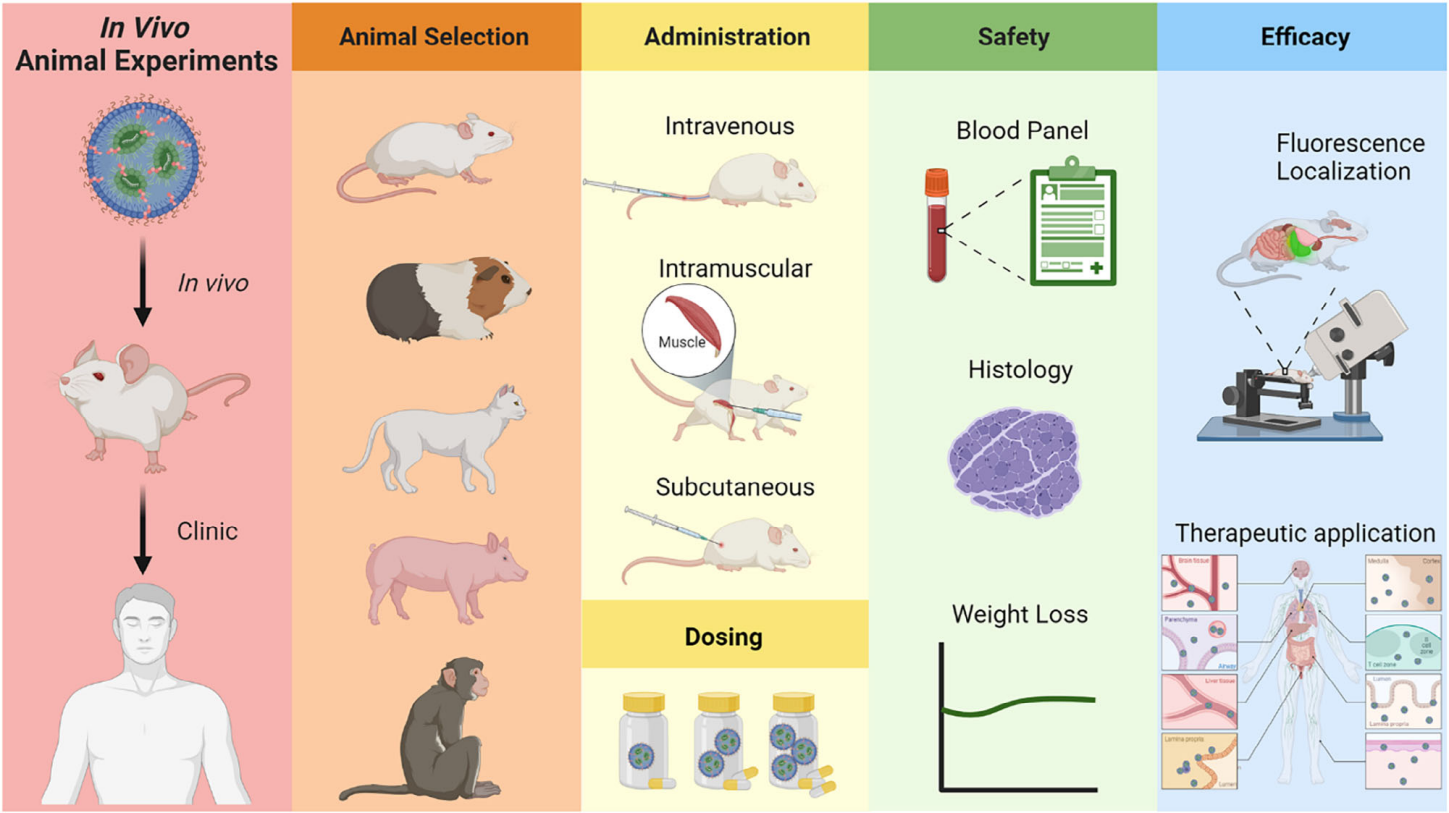
Key in vivo animal experiments of RNA‐based LNPs.

Evaluating RNA‐based LNP systems in vivo is a critical step in translating these promising delivery platforms into clinical trials. In vivo assessments provide valuable insights into the safety, efficacy, and therapeutic potential of LNP‐mediated RNA delivery within living organisms. In this perspective, we summarized some generalizable considerations to evaluate the in vivo efficacy of RNA‐based LNPs (Figure 4).

The first thing for in vivo animal experiments is to select a suitable animal model to ensure the relevance and translatability of the research. For example, mice are the commonly used animal species for preclinical research due to their high degree of genetic similarity with humans.^[^ ^87^ ^]^ In addition, they can also be easily genetically manipulated, allowing researchers to create transgenic, knockout, or knock‐in mouse models, which is particularly important for investigating RNA‐based therapies targeting specific genetic disorders.^[^ ^88^ ^]^

Animals can also be selected based on their applications, such as cancer, cardiovascular disorders, metabolic diseases, infectious diseases, and autoimmune diseases. Mice are commonly selected animals to study immune responses, as their immune systems share similarities with humans.^[^ ^89^ ^]^ Other applications, such as sensorineural hearing loss involve using guinea pigs and cats because they have more similar anatomical and physiological inner ear structures as humans compared to mice.^[^ ^90^, ^91^ ^]^ Moreover, they have a broader auditory range and are particularly sensitive to noise exposure, similar to humans. This sensitivity is important for studying noise‐induced hearing loss, understanding its mechanisms, and developing strategies for noise protection and prevention. While guinea pigs and cats offer distinct advantages for inner ear disease research, it's worth noting that the choice of animal model should rely on the specific research goals and the experimental requirements. In some cases, especially when specific genetic or molecular aspects are the focus, mice may also be used alongside these larger animal models. Pre‐clinical studies of RNA‐based LNPs are often required to be succeed on non‐human primates like monkeys before going into clinical trials. Other factors such as cost, maintenance, housing, and ease of handling also need to be considered for the selection of animals.

#### 2.4.2 Administration, dosing and safety

Administration routes and dosing for RNA‐based LNPs are essential aspects of designing and developing effective RNA‐based treatments. The choice of administration routes depends on the intended therapeutic effects and applications. General administrated strategies of RNA‐based LNPs include intravenous (IV) injection, intramuscular (IM) injection, and subcutaneous (SC) injection. Administration routes can impact the biodistribution of LNPs. IV injection is suitable for evaluating the biodistribution of the LNP platforms and understanding the main target tissues or organs. IM and SC injections are ideal for sustained and more controlled RNA release, often used in vaccine applications. Other administration routes, including intradermal, intravitreal, intranasal, intrathecal, and intratumor administration are locally administration routes that have been applied for specific applications.

The dosing of RNA‐based LNPs in animals is influenced by several factors. Animal size and body weight are the factors determining the LNP dose. Large animals typically require more RNA therapeutic doses compared to smaller ones. This dose is often calculated based on milligrams of overall RNA per kilogram of body weight (mg/kg). Researchers have used this dosing regimen to evaluate its in vivo efficacy.^[^ ^92^, ^93^, ^94^ ^]^ Furthermore, RNA dosing also relies on the pharmacokinetics of how the LNPs are distributed, absorbed, metabolized, and excreted, as well as target tissue characteristics such as volume and vascularization and the administration routes. For specific applications, like vaccines, it is suggested to dose RNA LNPs several times to generate longer‐lasting immunity.

Dose response studies are often performed to establish the safety profile of the LNP delivery systems because RNA‐based LNPs need to be within a safe range to minimize adverse effects. Safety parameters are very important to evaluate LNPs, as FDA requires rigorous safety data to approve their clinical applications. Several in vivo tolerability data are required. For example, the whole blood panel test and organ function, particularly the liver and kidneys, including alkaline phosphatase (ALP), alanine transaminase (ALT), aspartate transferase (AST), blood urea nitrogen (BUN), and creatinine (CREAT) levels, can be monitored between the LNPs treatment group and control group. Histological analysis can also be used to detect any tissue damage or inflammation after the treatment of LNPs. It is also suggested to monitor the animal weight change, as dramatic weight loss is also related to the high toxic effects of the formulated LNP platforms.

In summary, the choice of administration routes and dosing depends on the specific therapeutic goals and the anatomical or pathological factors associated with the target tissues or organs. By carefully selecting the appropriate dosing and administration route, researchers and clinicians can optimize the delivery of RNA‐based LNPs and enhance the therapeutic potential of these innovative treatments. As research in this field continues to advance, the development of more targeted and effective administration methods is likely to play a crucial role in the success of RNA LNP therapies for a wide range of diseases and conditions.

#### 2.4.3 In vivo therapeutic efficacy

The important part is to determine the in vivo efficacy of RNA LNPs in delivering and expressing RNA therapeutic drugs depending on their applications, such as mRNA vaccines, mRNA protein expressions, or RNA interference (RNAi) treatments. For mRNA vaccines or cancer immunotherapies, several efficacy parameters can be monitored, such as the immune response to the delivered antigen (e.g. a viral spike protein), antibody levels in the blood serum, CD4 or CD8 T cell responses, tumor size change, and the survival of the animals. To evaluate the efficacy of RNA‐based LNPs systems, FLuc mRNA is commonly used as RNA for a pilot study, as the FLuc protein level and the biodistribution of the target organ can be easily quantified and monitored via IVIS image. For mRNA therapeutic generating secreted protein, such as enzymes, ELISA can be used to quantify the protein level in the blood serum to evaluate the in vivo efficacy of the LNP systems. Building on those pilot results, the LNP system with the best performance can be further investigated for therapeutic models. In the cases of RNAi therapies such as siRNAs or short hairpin RNAs (shRNAs), successful LNP systems can inhibit specific target genes and further affect the production of target proteins, for example, suppressing tumor growth in cancer therapies.^[^ ^95^, ^96^ ^]^ Additionally, LNPs encapsulating mRNA encoded with specific growth factors can be employed in tissue engineering to stimulate the growth and differentiation of the tissues.

Overall, in vivo animal experiments are instrumental in bridging the gap between promising RNA‐based LNP delivery systems and their clinical applications. These studies provide critical data on safety, efficacy, and the potential application of RNA‐based LNPs to treat a wide range of diseases, such as genetic disorders, infectious diseases, and cancer. By conducting rigorous in vivo evaluation, researchers can optimize these RNA delivery systems and progress toward clinical translation.

## 3 CONCLUSIONS AND OUTLOOKS

In conclusion, this perspective represents a pivotal and ever‐evolving process that lies at the forefront of modern nanomedicine and RNA drug delivery. We provide a perspective for studying the performance of an RNA‐based LNP drug delivery platform that encompasses a range of critical stages, from formulation and characterization development to in vitro and in vivo evaluation. The formulation provides the foundation of LNP development, which involves the careful selection of lipid components and formulation strategies, as well as the encapsulation of RNA molecules. This stage is vital in determining the stability and efficiency of the resulting LNPs. Researchers must also balance factors like particle size, surface charge, and RNA EE to create LNPs with the desired characteristics for successful delivery. Moving forward to in vitro evaluation, researchers should evaluate RNA‐based LNPs in aspects including cell viability, cellular association/uptake, intracellular trafficking and endosomal escape, and transfection (such as protein expression level) in either a dose‐responsive or time‐dependent fashion. These experiments provide a critical understanding of how the LNPs function at the cellular level. It is during this phase that fine‐tuning of the LNP formulation and optimization of efficacy protocols occur, enhancing the likelihood of a successful in vivo evaluation. Preclinical in vivo studies serve as the bridge between in vitro experiments and human clinical trials. These investigations allow for the evaluation of therapeutic efficacy, biodistribution, pharmacokinetics, and toxicology. The selection of appropriate animal models is also an important step, as it aids in predicting the potential outcomes and safety profile of RNA‐based LNPs. Importantly, it is during these studies that researchers can assess the effects of multiple doses and gather critical data for regulatory submissions. Moving forward, the systems can then translate from bench to clinical trials in three phases. Phase I trials are focused on safety, dosage, and initial efficacy. Phase II trials aim to further establish therapeutic potential. Phase III trials provide a larger‐scale assessment of the therapy's effectiveness in diverse patient populations. Successful completion of these phases could pave the way for regulatory approval and eventual patient access to RNA‐based LNPs. This perspective is to provide useful guidance for new researchers who want to work in the RNA‐based LNP field, with the ultimate goal of selecting the RNA‐based LNPs with the best performance and transferring them into clinical trials.

Looking forward, the outlook for RNA‐based LNPs is exceptionally promising. We are witnessing the rapid expansion of these technologies across various therapeutic domains. mRNA vaccines, which have proven their effectiveness against COVID‐19, have evolved in vaccine development for other pathogens and even cancer immunotherapies. RNAi therapies, capable of precisely silencing disease‐causing genes, hold the potential to transform the landscape of genetic and rare diseases. Moreover, ongoing research is exploring the versatility of RNA‐based LNPs, with applications in regenerative medicine, personalized therapies, as well as emerging fields like CRISPR‐based gene editing. Further advances in RNA‐based LNP technology, including enhanced stability, tissue‐specific targeting, and controlled release mechanisms, will unlock even greater therapeutic potential. As the regulatory pathway for these therapies becomes more well‐defined and streamlined, the translation of groundbreaking research from the laboratory to the clinic is expected to become increasingly efficient. As the field of RNA‐based therapeutics continues to evolve, this perspective will remain at the forefront of innovation, driving the development of groundbreaking treatments for a wide range of diseases and disorders.

## CONFLICT OF INTEREST STATEMENT

The authors declare no conflicts of interest.
